# Molecular identification of some wild Nigerian mushrooms using internal transcribed spacer: polymerase chain reaction

**DOI:** 10.1186/s13568-018-0661-9

**Published:** 2018-09-20

**Authors:** Mobolaji Adeniyi, Yinka Titilawo, Anthonia Oluduro, Olu Odeyemi, Motebang Nakin, Anthony Ifeanyi Okoh

**Affiliations:** 1 Department of Microbiology, Obafemi Awolowo University, Ile-Ife, Nigeria; 2 Department of Microbiology, Osun State University, Osogbo, Nigeria; 3 Department of Biology/Microbiology/Biotechnology, Faculty of Science, Federal University, Ndufu-Alike, Ikwo, Nigeria; 4 Risk and Vulnerability Science Centre, Walter Sisulu University, Mthatha, 5099 South Africa; 5 SAMRC Microbial Water Quality Monitoring Centre, University of Fort Hare, Alice, 5700 South Africa

**Keywords:** Mushrooms, ENPOST, Molecular identification, Food security, Nigeria

## Abstract

Identification of fungal species based on morphological characteristics is tedious, complex, prone to errors, and thus cannot be completely relied upon. In this study, internal transcribed spacers (ITS 1 and 4)—polymerase chain reaction was employed to amplify DNA of 19 mushroom isolates collected at Environmental Pollution Science and Technology farm, Ilesa, Southwest Nigeria. The PCR amplification of ITS1 and 4 of the mushrooms isolates yielded approximately 850 bp. Amplicons obtained were sequenced and identified using BLASTn in the NCBI. The BLASTn results revealed that *Termitomyces aurantiacus* (3), *Tricholoma matsutake* (8), *Tricholoma robustum* (2), *P*. *ostreatus* (4), *Schizophyllum commune* (1) and *Pleurotus pulmonarius* (1) were fully represented. Only *Tricholoma matsutake* (KT273371), *Pleurotus pulmonarius* (KY962469) and *Tricholoma matsutake* (AF438605) had 100% similarity with reference strain. However, the phylogenetic analysis of the isolates showed low genetic relatedness with reference strains. This study revealed the novelty of the mushroom strains and thus advocating the need for strict conservation measures and further investigations on their potential benefits to mankind.

## Introduction

Mushrooms are regarded as visible fungi with distinctive carpophores (basidiocarps or fruiting bodies) which represent the reproductive stage in the life cycle of Basidiomycetes and some Ascomycetes (Das 2010). Morphologically, they are classified as puffballs, stinkhorns, bracket and gilled fungi, and may either be edible, inedible or poisonous (Bates 2006). They are usually abundant during the wet season in most countries of the world and have been found thriving on different substrates (Rogers 1994; Straatsma et al. 2001; LaRochelle and Berkes 2003; Gbolagade et al. 2006; Djelloul and Samraoui 2011; Dwivedi et al. 2012; Pushpa et al. 2012; Andrew et al. 2013; Vyas et al. 2014; Rashid et al. 2016; Rumainul and Aminuzzaman 2016).

Generally, macrofungi are regarded as important bioresource because of their diverse ecological, nutritional, health and medicinal benefits (Odeyemi et al. 2014). They are decomposers of dead organic matters especially those containing lignin and cellulose, and also aid nutrient recycling in the environment (Nilsson et al. 2011). In addition, they are rich in protein, crude fibre, vitamins and minerals, and possess medicinal benefits including anticholesterol, antitumor, antimicrobial, antiviral, antineoplastic, antimutagenic, antioxidant, antilipidemic, antidiabetic antihyperglycaemic, antihypotensive, antiparasitic, anti-inflammatory hepatoprotective, hypocholesterolemic, immunodulatory and anti-ageing properties (Borchers et al. 2008; Rathee et al. 2011; Patel et al. 2012; Duru and Çayan 2015).

Accurate taxonomy is essential for exploitation of the numerous advantages an organism offers (Odeyemi et al. 2014). Before now, traditional method of mushroom identification is based on the size, shape and margin of carpophore, length, diameter, location and texture of stipe, the spore-bearing surface, habitat, habit, season they appear, spore prints, use of coloured photographs, and any other unique feature present on the fruiting body (FAO 2006; Odeyemi and Adeniyi 2015). However, mutation resulting in genetic changes and which may not be expressed, and the indistinctive nature of mushroom mycelial forms remains a setback with taxonomy using morphological characteristics (Lian et al. 2008; Appiah et al. 2017).

Modern molecular technique reduces the challenges of inconspicuous nature, inconsistent morphology and indiscrimination among fungal species often associated with traditional method of nomenclature (Blackwell et al. 2006; Nilsson et al. 2011). This procedure employs the use of genetic marker identifiable to a specific region of either the coding or non-coding portion of the fungal genome, usually the nuclear encoded ribosomal RNA (rRNA). These genes present in multiple copies and contain conserved coding (small subunit—SSU and large subunit—LSU) as well as variable non-coding parts (internal transcribed spacers—ITS) are highly conserved within a species (Cui et al. 2011). In addition, they are suitable for developing genetic probes to classify fungi and also reveal their phylogenetic relationship (Cui et al. 2011; Siddiquee et al. 2015).

In China, PCR-based approach was employed to identify *Boletus edulis* and *Verpa bohemica* using rDNA ITS sequences (Lian et al. 2008; Anand and Chowdhry 2013). In addition, eight wild mushrooms belonging to the genera *Amanita*, *Astraceus*, *Termitomyces* and *Volvariella* were characterized in the region (Das et al. 2013). Similar identification was performed on *Boletus edulis*, *B. aestivalis*, *B. luridus*, *Amanita muscaria* and *Hebeloma radicosum* in Italy (Iotti et al. 2005) and *Tricholoma giganteum* and *Calocybe indica* isolates in Bangalore (Pushpa et al. 2012). Likewise, *Agaricus bisporus* white and brown varieties, *Pleurotus sajor*-*caju*, *Pleurotus eryngii*, *Lentinula edodes* and *Flammulina velutipes* were identified in Malaysia (Avin et al. 2014) and *Amanita* sp. in India (Zhang et al. 2004).

In the black African countries, studies on mushrooms taxonomy using the molecular technique have not been widely investigated. In Kenya, Ojwang (2012) characterized seventy-one *Pleurotus* species collected from the wild whereas in Nigeria, literatures revealed that Oyetayo (2009, 2014), Bankole and Adekunle (2012) and Awala and Oyetayo (2015) have identified mushrooms. The samples were collected from Ekiti, Lagos, Ondo and Oyo states of the Southwestern Nigeria and characterized using the internal transcribed spacer—polymerase chain reaction ITS-PCR). Diversity of mushrooms abounds at ENPOST farm, Ilesa, Osun State, Southwest Nigeria. However, paucity of information regarding their taxonomy using genetic approach greatly limits optimal exploitation for diverse human benefits. To the best of our knowledge, this is the first of its kind in the region and it is in the light of this we aimed at investigating the variability of some wild mushrooms collected at the ENPOST farm, Ilesa, Southwest Nigeria using ITS regions.

## Materials and methods

### Description of study area

Environmental Pollution Science and Technology (ENPOST) farm, Ilesa, Southwest Nigeria is located between (Latitude 4°42′30″E to 4°42′45″E longitude 7°36′55″N to 7°37′10″N). The farm which spans about 10 hectares was established primarily to address the challenges of environmental pollution, food insecurity and agroforestry/biodiversity destruction and provide opportunities for research on natural resources.

### Sample collection and presumptive identification

Nineteen mushroom samples were randomly collected at ENPOST farm, Ilesa, Southwest Nigeria. The mushrooms were from decaying woods and leaves, soil debris and termite mounds from different locations within the farm over a 2 year sampling regime (April 2014 and March 2016). Samples were harvested from their respective substrate, placed in different labelled sterile paper bags and immediately taken to the laboratory for further analysis. The samples were kept in the refrigerator (4 °C) before analysis. Presumptive identification was based on the morphology of the mushrooms previously described (Rogers 1994; Nwordu et al. 2013).

### Tissue culture of mushroom fruiting bodies

Fruiting bodies collected from the wild were gently cleaned with water, the undercap surface (gills) cut into small sizes (1 cm^2^) and surface sterilized in 70% ethanol for 30 min. The surface sterilized mushroom pieces were aseptically placed on sterile potato dextrose agar (PDA) plates and incubated for 7–10 days. Sub-culturing of mushroom mycelia was done and pure isolates were preserved on PDA slant at 4 °C until when needed.

### Extraction and PCR amplification of genomic DNA

Total genomic DNA was extracted from a 5–7 day old mycelial mat using the ZR Fungal/Bacterial DNA kit™ (Zymo Research, USA) following the manufacturer instructions. Briefly, 100 mg wet weight of mushroom mycelium suspended in 200 µl phosphate buffer solution buffer was added to a ZR BashingBead ™ lysis tube and vortexed at 10,000 rpm for 5 min. Thereafter, the ZR BashingBead ™ lysis tube was centrifuged at 1000 rpm for 1 min, 400 µl of the supernatant transferred into a Zymo-Spin™ IV spin filter in a collection tube and centrifuged at 7000 rpm for 1 min. Exactly 1200 µl of Fungal/Bacterial DNA binding buffer was added to the filtrate and 800 µl of the mixture was centrifuged twice at 10,000 rpm for 1 min. In a new Zymo-Spin™ IIC column, 200 µl of DNA pre-wash buffer and 500 µl Fungal/Bacterial DNA wash buffer was added and centrifuged at 10,000 rpm for 1 min respectively. The column was then transferred into a clean 1.5 ml microcentrifuge tube, after which 100 µl DNA elution buffer was added directly into the column matrix and centrifuged at 10,000 rpm for 30 s to elute the DNA. Ultra-pure DNA was stored at − 80 °C for further use.

The internal transcribed spacer (ITS) region of the rDNA was amplified by PCR with previously described universal primers ITS1 (5′-TCC GTA GGT GAA CCT GCG G-3′) and ITS4 (5′-TCC TCC GCT TAT TGA TAT GC-3′) (White et al. 1990). PCR reaction mixture was performed in a total volume of 50 μl containing 30–50 ng DNA, 100 mM of each primer, 0.05 U/μl *Taq* DNA polymerase, 4 mM MgCl_2_, and 0.4 mM of each dNTP. The amplification reaction was done with a C1000 Touch thermal cycler (BioRad, USA). Method of Das et al. (2013) with slight modifications were employed for thermal cycling conditions. Initial denaturation at 95 °C for 5 min, followed by 30 cycles of denaturation at 95 °C for 1 min, annealing at 57 °C for 1 min and extension at 72 °C for 2 min and a final extension at 72 °C for 7 min.

The PCR amplicons were analysed by electrophoresis. Five microliters of DNA ladders (1 kb) and 7 μl of the samples were loaded in wells of agarose gel (1% w/v containing ethidium bromide) and allowed to run at 60 V for 2 h. Gel results were visualized with a ChemiDoc™ MP System (Bio-Rad Laboratories, Hercules, CA, USA) to confirm the expected size of the product. The remaining PCR products were purified using NucleoSpin Gel and PCR Clean-up kit (Macherey–Nagel, Germany) (Aremu and Babalola 2015).

### DNA sequencing and ITS region analysis

The sanger sequencing of the purified PCR products was done at Inqaba Biotechnical Industrial (Pty) Ltd, Pretoria, South Africa with PRISM™ Ready Reaction Dye Terminator Cycle sequencing kit using the dideoxy chain termination method and electrophoresed with a model ABI PRISM^®^ 3500XL DNA Sequencer (Applied Biosystems, Foster City, CA, USA) following manufacturer’s instructions.

For good quality sequence assurance, ChromasLite version 2.33 software was used for the analysis of chromatograms (sense and antisense) resulting from sequencing reaction (Technelysium 2004). The resulting chromatograms were edited using BioEdit Sequence Alignment Editor (Hall 2004). After this, the resulting consensus 16S rDNA sequences obtained were blasted in the NCBI (http://www.ncbi.nlm.nih.gov) database with the BLASTn for homology in order to identify the probable organism in question (Altshul et al. 1997). The sequences were deposited in the GenBank.

### Phylogenetic analysis

The phylogenetic analyses based on the 16S rDNA gene were further used to characterize the organisms in order to establish relationships among them. The partial 16S rDNA sequences obtained were utilized in the search of reference nucleotide sequence available in NCBI GenBank database using BLASTn algorithm (Altshul et al. 1997). Mafft version 7.0 was employed in the multiple alignment of nucleotide sequences (Katoh and Toh 2010) while trees were drawn based on character based method (Maximum Likelihood) for comparing set of data against set of models of evolution using MEGA 7 (Kumar et al. 2016). Putative chimeric sequences were identified using the Chimera Buster 1.0 software. Manipulation and tree editing were carried out using TreeView (Page 1996).

### Data analysis

Statistical analysis was performed using IBM Statistical Package for Social Sciences [(SPSS) Version 22 software]. The one way ANOVA was executed to investigate the possible existence of correlation between the presumptively identified wild mushroom and counts obtained with respect to the 3 year sampling duration. Correlations and test of significance were considered statistically significant when P values were < 0.05.

## Results

### Presumptive identification

**Fig. 1:**
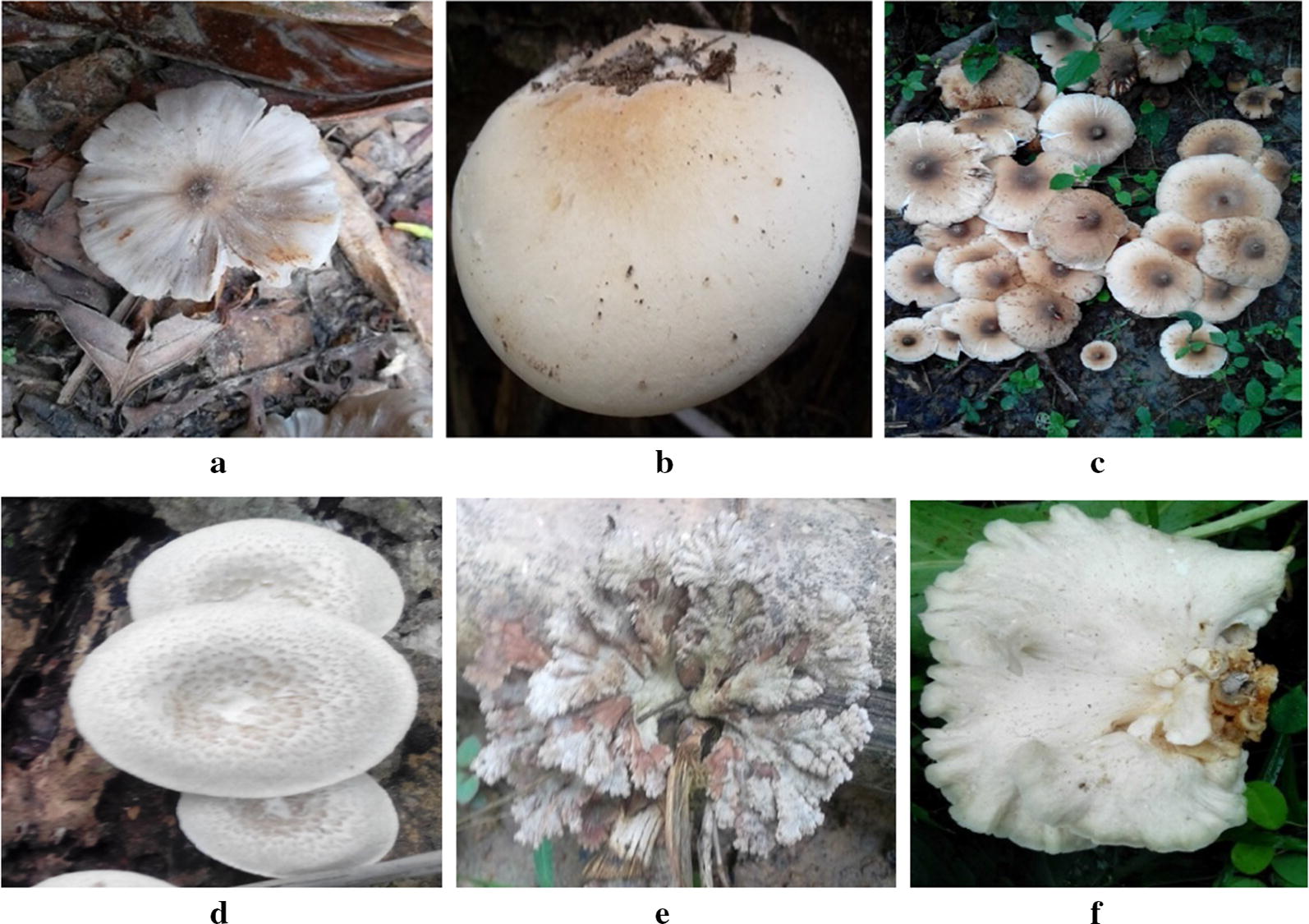
Representative pictures of the different mushrooms. **a** *Tricholoma matsutake*, **b** *Tricholoma robustum*, **c** *Termitomyces aurantiacus*, **d** *Pleurotus ostreatus*, **e** *Schizophyllum commune* and **f** *Pleurotus pulmonarius*

The morphological characteristics of the 19 mushrooms indicated that they belonged to four genera namely, *Tricholoma*, *Termitomyces*, *Schizophyllum* and *Pleurotus* (Table 1). Eight (8) of the mushrooms were presumptively identified as *Tricholoma matsutake*, *Tricholoma robustum* (2), *Termitomyces aurantiacus* (3), *Pleurotus ostreatus* (4), *Schizophyllum commune* (1), *Pleurotus pulmonarius* (1). Representative pictures are in Fig. 1.

**Table 1:** Growth substrates and counts of wild mushrooms

Sample	Presumptive identification	Growth substrate	Year		
			2014	2015	2016
F1	*Tricholoma matsutake*	Soil debris	7	2	1
F2	*Termitomyces aurantiacus*	Termite mound	53	23	48
F3	*Termitomyces aurantiacus*	Termite mound	65	38	52
F4	*Termitomyces aurantiacus*	Termite mound	35	17	36
F5	*Tricholoma matsutake*	Soil debris	3	1	2
F6	*Tricholoma matsutake*	Soil debris	1	0	3
F7	*Schizophyllum commune*	Dead *Bambusa vulgaris* log	2530	1431	2321
F8	*Pleurotus pulmonarius*	Dead *Mangifera indica* log	63	12	27
F9	*Tricholoma robustum*	Soil debris	7	3	1
F10	*Tricholoma matsutake*	Soil debris	5	2	4
F11	*Tricholoma matsutake*	Soil debris	4	3	4
F12	*Pleurotus ostreatus*	Dead *Elaeis guineensis* log	17	6	13
F13	*Pleurotus ostreatus*	Dead *Elaeis guineensis* log	13	9	8
F14	*Pleurotus ostreatus*	Dead *Elaeis guineensis* log	6	12	16
F15	*Pleurotus ostreatus*	Dead *Elaeis guineensis* log	21	8	9
F16	*Tricholoma matsutake*	Soil debris	3	0	0
F17	*Tricholoma robustum*	Soil debris	5	0	3
F18	*Tricholoma matsutake*	Soil debris	7	2	5
F19	*Tricholoma matsutake*	Soil debris	4	3	6

Interestingly, each species was obtained from the same growth substrate from different locations of the farm. Generally, fruiting bodies of the mushroom species were frequent in 2014 and *Schizophyllum commune* dominated throughout the sampling period.

Overall, the statistical one way ANOVA revealed a significant difference in the counts of *Schizophyllum commune* compared to other mushroom samples (P < 0.05), whereas the differences in the counts of *Tricholoma matsutake, Termitomyces aurantiacus, T. robustum, Pleurotus pulmonarius, P. ostreatus* were not statistically significant over the sampling regime (P > 0.05).

### PCR amplification

**Fig. 2:**
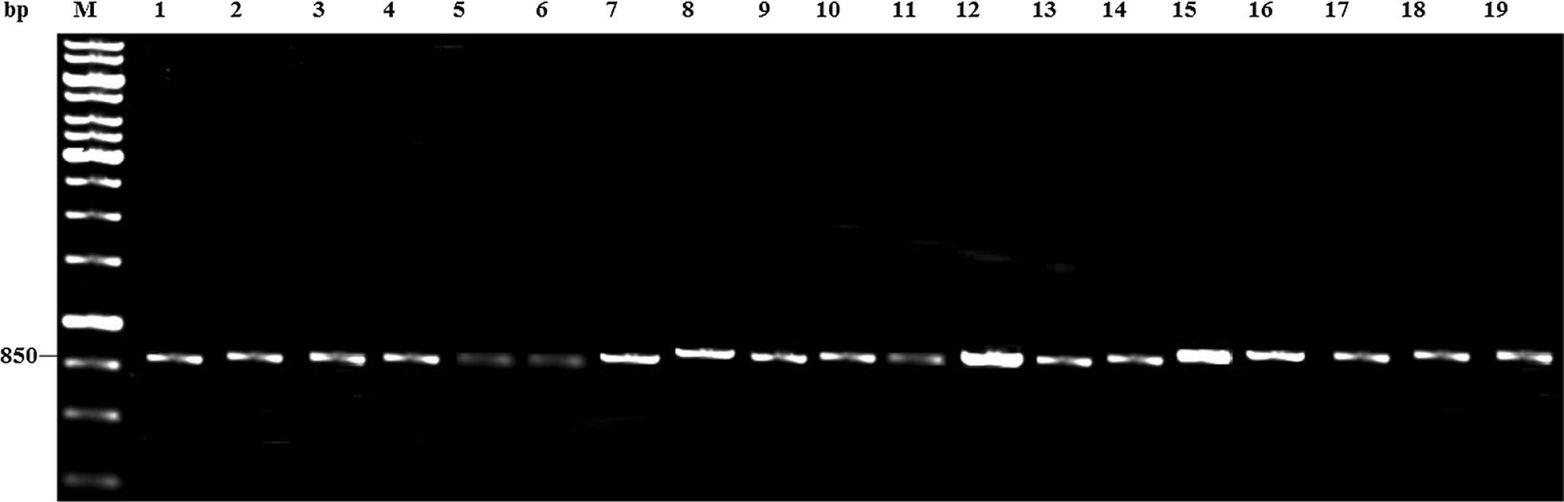
Agarose gel photograph amplified DNA sequences of 850 bp. Lane M = 1 kb molecular weight marker; Lane 1—*Tricholoma matsutake*; Lane 2—*Termitomyces aurantiacus*; Lane 3—*Termitomyces aurantiacus*; Lane 4—*Termitomyces aurantiacus*; Lane 5—*Tricholoma matsutake*; Lane 6—*Tricholoma matsutake*; Lane 7—*Schizophyllum commune*; Lane 8—*Pleurotus pulmonarius*; Lane 9—*Tricholoma robustum*; Lane 10—*Tricholoma matsutake*; Lane 11—*Tricholoma matsutake*; Lane 12—*Pleurotus ostreatus*; Lane 13—*Pleurotus ostreatus*; Lane 14—*Pleurotus ostreatus*; Lane 15—*Pleurotus ostreatus*; Lane 16—*Tricholoma matsutake*; Lane 17—*Tricholoma robustum*; Lane 18—*Tricholoma matsutake*; Lane 19—*Tricholoma matsutake*

The PCR amplification of ITS 1 and 4 of the 19 mushrooms yielded approximately 850 bp (Fig. 2).

### DNA sequencing analysis

The BLASTn results revealed that *Tricholoma matsutake* dominated (8 out of 19) the samples collected. Two (2) *Tricholoma robustum*, three (3) *Termitomyces aurantiacus*, four (4) *P*. *ostreatus* were identified whereas one (1) *Schizophyllum commune* and *Pleurotus pulmonarius* were represented. All the *T*. *aurantiacus* were closest to GU594650 with percentage similarity ranging between 77 and 90%. While five of the *Tricholoma matsutake* identified with KJ937003, percentage similarity ranging between 85 and 99%, the remaining three agreed with JF440958 in 98%, and KT273371 and AF438605 both in 100% respectively. The *Pleurotus ostreatus* species identified with 4 different strains with percentage identity > 80%. *Schizophyllum commune* and *Pleurotus pulmonarius* were similar to strains KY962469 and KX394806 respectively (Table 2).

**Table 2:** Summary of BLASTn results

Sample	BLASTn identity of sample	Percentage (%) identity	Accession number
F1	*Tricholoma matsutake* (KT273371)	100	MF037408
F2	*Termitomyces aurantiacus* (GU594650)	90	MF037409
F3	*Termitomyces aurantiacus* (GU594650)	79	MF037410
F4	*Termitomyces aurantiacus* (GU594650)	77	MF037411
F5	*Tricholoma matsutake* (KJ937003)	93	MF037412
F6	*Tricholoma matsutake* (KJ937003)	85	MF037413
F7	*Schizophyllum commune* (KX394806)	99	MF037414
F8	*Pleurotus pulmonarius* (KY962469)	100	MF037415
F9	*Tricholoma robustum* (AF455529)	90	MF037416
F10	*Tricholoma matsutake* (AF438605)	100	MF037417
F11	*Tricholoma matsutake* (KJ937003)	99	MF037418
F12	*Pleurotus ostreatus* (KC582642)	94	MF038192
F13	*Pleurotus ostreatus* (FJ224121)	93	MF037419
F14	*Pleurotus ostreatus* (GQ249947)	98	MF037420
F15	*Pleurotus ostreatus* (EU622253)	80	MF037421
F16	*Tricholoma matsutake* (JF440958)	98	MF037422
F17	*Tricholoma robustum* (AF455529)	90	MF037423
F18	*Tricholoma matsutake* (KJ937003)	84	MF038193
F19	*Tricholoma matsutake* (KJ937003)	97	MF037424

### Phylogenetic analysis

**Fig. 3:**
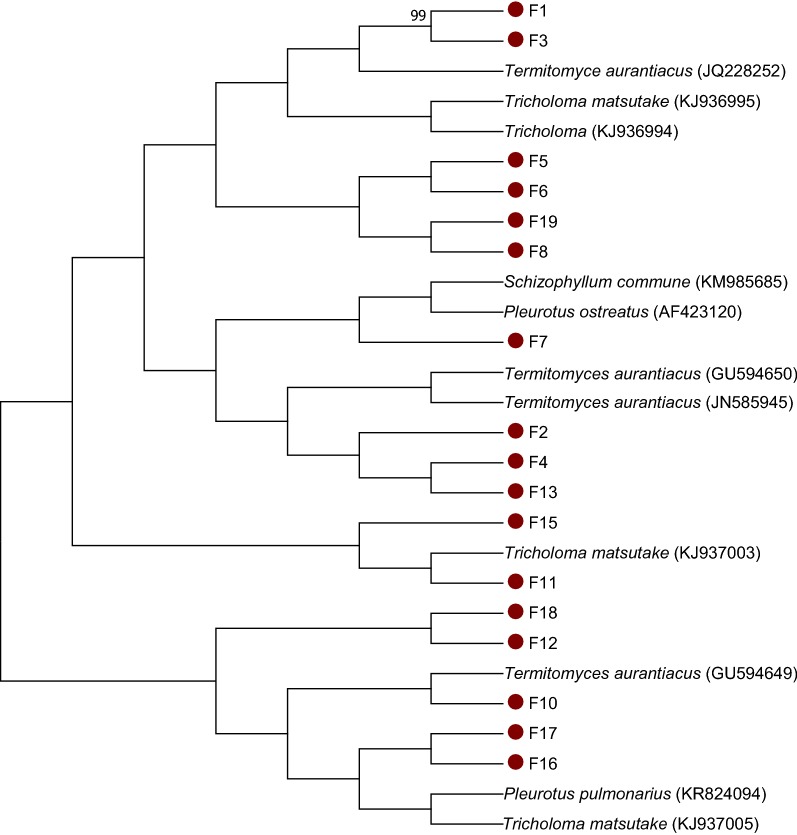
Phylogenetic analysis based on partial 16S rDNA sequences of 19 wild mushrooms obtained in this study and 11 other reference sequences from the GenBank. The tree was reconstructed using maximum likelihood method as implemented in Mega 7

The phylogenetic analysis revealed that isolates F10, F12, F16, and F17 and F18 clustered with *Termitomyces aurantiacus* (GU594649) *Pleurotus pulmonarius* (KR824094) and *Tricholoma matsutake* (KJ93005) respectively. Also, isolates F11 and F15 clustered with *Tricholoma matsuake* (KJ937003) (Fig. 3). In the same manner, F2, F4, F13 exhibited similarity with *Termitomyces aurantiacus* and F7 with *Schizophyllum commune* (KM985685) and *Pleurotus ostreatus* (AF423120). Likewise, F1 and F3, F5, F6, F8, F19 clustered with *Tricholoma* (KJ936994)*, Tricholoma matsutake* (KJ936995) and *Termitomyces aurantiacus* (JQ228252) respectively.

## Discussion

Accurate taxonomy is essential for further studies on any organism. Usually, traditional method of mushroom identification is based on the size, shape and margin of carpophore, length, diameter, location and texture of stipe, the spore-bearing surface, habitat, habit, season they appear, spore prints, use of coloured photographs, and any other unique feature present on the mushroom fruitbody (Food and Agricultural Organization (FAO) 2006; Odeyemi et al. 2014). Morphological characteristics identified the mushrooms as *Tricholoma matsutake*, *Tricholoma robustum*, *Termitomyces aurantiacus*, *Pleurotus ostreatus*, *Schizophyllum commune* and *Pleurotus pulmonarius*. However, this method cannot be completely relied on and also, characterization using mushroom mycelial form are challenging because they cannot be easily differentiated using either morphological characteristics or organic/inorganic components (Lian et al. 2008).

Several factors including substrate availability, suitable moisture and temperature influence the growth of mushrooms in the wild (Nwordu et al. 2013; Tedersoo et al. 2014). In the study, frequency of mushrooms was generally higher in 2014. Thus, favourable environmental conditions favouring growth of mushrooms exist in the year. The dominance of *Schizophyllum commune* throughout the sampling period may be due to its small size and abundance of supporting growth substrates.

Genetic identification is an essential diagnostic tool for unraveling the rich biodiversity of wild mushrooms (Rajaratnam and Thiagarajan 2012). Importantly, the ITS region of nuclear ribosomal DNA, including ITS1, 2 and 4, has been used to validate the inconsistency in macrofungi taxonomy at the species and subspecies level (Cui et al. 2011; Raju et al. 2014; Appiah et al. 2017). In Nigeria, molecular identification of mushrooms using the ITS region is scarcely studied.

In the present investigation, PCR product of mushrooms (approximately 850 bp) disagrees with Appiah et al. (2017) who reported 400 and 600 bp for *P*. *ostreatus* and *S. commune* respectively using ITS 1 and 4. *Tricholoma robustum* was previously reported to fall in the range of 450 and 650 bp (Apollos et al. 2017). The difference in band sizes may be due to variations in the quality of DNA used in PCR (Lee et al. 2012; Lorenz 2012; Kim et al. 2016). Albeit, all the fragment sizes fall within the range (350–880 bp) reported by Fujita et al. (2001) for fungi using ITS 1 and 4. Similarly, Siddiquee et al. (2015) reported approximately 500 bp for *T. aurantiacus* using ITS 1 and 2. The variability of fungal ITS region and difference in primer combination used in amplification may be responsible for this (Fujita et al. 2001; Gomes et al. 2002; Krimitzas et al. 2013). Paucity of information however exists on the product size of *P. pulmonarius*, *T. matsutake* and *T. robustum* using ITS regions.

Interestingly, the sequence analysis of this work corroborates Appiah et al. (2017) who ascertained the identity of *P. ostreatus *and *S. commune* from central region of Ghana with 98 and 100% similarity. Also, Siddiquee et al. (2015) confirmed the identity of *T. aurantiacus* from four different termite mounds from Seriserdang area and reported 100% similarity stating that they were ex-strains of *T. aurantiacus* GU594650, JN585945, JN585945 and JQ228252 from GenBank repository. Also, *P. pulmonarius* had previously been identified in Malaysia using ITS 1 and 2 (Avin et al. 2014). In this study, most of the gene sequences of mushrooms indigenous to Nigeria are not 100% homology with existing gene sequence found in NCBI GenBank. Oyetayo 2014, stated that differences in the gene sequences maybe due to the different ecological zones where the mushrooms are present.

Low similarity value expressed by isolates with the reference taxa belonging to different species result in DNA reassociation that rise above the 70% threshold values (Stackebrandt et al. 2002). In the present study, phylogenetic analysis of the isolates showed low genetic relatedness with reference strains. Thus, the mushroom isolates are quite different from the reference sequences from the Genbank. This suggests that the similarity shared can be wiped off after sometimes due to environmental conditions (Konstantinidis and Stackebrandt 2013). In addition, isolates did not cluster with the reference taxa from the GenBank as a result of distinct nucleotide signature (Togashi et al. 2001; Aremu and Babalola 2015). From the phylogenetic distinctness point of view, these fungal isolates are probably novel mushroom species, and therefore calls for stern safeguarding and further investigations on the associated benefits.

## Abbreviations

ITS: internal transcribed spacers
NCBI: National Center for Biotechnology Information
ENPOST: Environmental Pollution Science and Technology
FAO: Food and Agriculture Organization
DNA: deoxyribonucleic acid
rDNA: recombinant deoxyribonucleic acid
SSU: small subunit
LSU: large subunit
PCR: polymerase chain reaction
USA: United States of America
MgCl_2_: magnesium chloride
dNTP: deoxyribonucleotide triphosphate
BLAST: basic local alignment search tool
MEGA: molecular evolutionary genetics analysis
IBM: International Business Machines
SPSS: statistical package for the social sciences
ANOVA: analysis of variance
